# STABILITY AND CHANGE IN DEPRESSIVE SYMPTOMS AND COGNITION FOR CENTENARIAN COHORTS

**DOI:** 10.1093/geroni/igz038.2423

**Published:** 2019-11-08

**Authors:** Gina Lee, Peter Martin

**Affiliations:** Iowa State University, Ames, Iowa, United States

## Abstract

The purpose of the study was to examine the stability and change of cognition and depression levels and how they predict each other over time. Participants of the Health and Retirement Study who survived to centenarian status (N = 331) were included in this study. The total cognition summary score and the CES-D summary score of depressive symptoms were used to conduct four cross-lagged regression analyses from wave 2 to wave 6. Age was used as a covariate. Results indicated that the stability coefficients for depressive symptoms and cognition from wave 2 to wave 6 were high. Depressive symptoms at wave 2 significantly predicted change in cognition at wave 3, whereas depressive symptoms at all other waves did not predict change in cognition in the next wave. Cognition did not predict changes in depressive symptoms for any wave. Age as a covariate predicted change in cognition in each following wave, particularly from wave 2 to wave 5. The coefficients without stability for depressive symptoms and cognition from wave 2 to wave 5 predicted each other significantly over time, except for the last wave. In conclusion, cognition and depressive symptoms predict each other over time, but they do not predict each other if stabilities are included in the analyses. Further research needs to examine the stability and change in depressive symptoms and cognition including more waves in order to examine whether cross-lagged effects fade or continue in very late life.

